# Obesity and risk of placenta accreta spectrum: A meta-analysis

**DOI:** 10.1515/med-2024-1047

**Published:** 2024-10-18

**Authors:** Ensiyeh Jenabi, Roya Najafi-Vosough, Arshia Nazari

**Affiliations:** Mother and Child Care Research Center, Hamadan University of Medical Sciences, Hamadan, Iran; Research Center for Health Sciences, Hamadan University of Medical Sciences, Hamadan, Iran; Student of Medicine, Dezful University of Medical Sciences, Khuzestan, Iran

**Keywords:** placenta accreta spectrum, obesity, meta-analysis, pregnancy

## Abstract

**Background:**

Some studies have indicated a notable association between obesity and placenta accreta spectrum (PAS), while others have not reported. Hence, we performed a meta-analysis to explore the association between obesity and the risk of PAS.

**Methods:**

To explore the association between obesity and PAS through observational studies, we conducted a systematic search across PubMed, Web of Science, Google scholar, and Scopus databases up to March 30, 2024. The meta-analysis utilized a random-effect model, with the quality of included studies assessed using the Newcastle–Ottawa scale. A significance level of less than 0.05 was considered statistically significant using Stata software, version 14 (StataCorp, College Station, TX, USA).

**Results:**

The association between obesity and PAS risk in crude studies showed significance (1.51 [95% CI: 1.19, 1.82; *I* ^2^ = 0.0%]). However, in adjusted studies, the association was not significant (1.25 [95% CI: 0.45, 2.05; *I* ^2^ = 52.0%]).

**Conclusion:**

These findings suggest that obesity has been proposed as potentially associated with a higher risk of PAS, particularly evident in crude studies. However, it is imperative to conduct prospective cohort studies with a large sample size and meticulous control of confounding variables to further elucidate this relationship.

## 1 Introduction

Histologically, placenta accreta spectrum (PAS) is characterized by either total or partial absence of decidua, accompanied by placental invasion into the myometrium [1]. PAS has emerged as a leading cause of peripartum hysterectomy, posing significant maternal morbidity and, in severe cases, maternal mortality on a global scale [2]. According to a meta-analysis, PAS prevalence ranges from 0.01 to 0.1% of deliveries [3]. The precise pathogenesis remains elusive. Potential causes include mechanical factors (such as primary decidua deficiency due to localized uterine wall trauma), biological factors (such as abnormal maternal responses to trophoblast invasion), or a combination of both mechanisms [4,5].

PAS disorder represents a grave maternal complication, posing a life-threatening condition [6]. Its gravity is underscored by the elevated risks of maternal and fetal mortality [7]. Maternal morbidity and mortality may result from profound hemorrhage, occasionally reaching life-threatening levels, frequently requiring blood transfusions [8]. Notably, PAS stands out as a contributor to postpartum hemorrhage, often culminating in hysterectomy [9].

The factors independently associated with PAS disorders were BMI ≥30, previous uterine surgery, previous postpartum hemorrhage, a higher number of prior cesareans, a placenta previa [10], multiple gestation, hypertensive disorders, *in vitro* fertilization, and smoking [11,12]. Conversely, hypertension disorders, low socioeconomic status, and carrying a male fetus have been identified as protective factors against PAS [11].

Obesity increases the likelihood of various diseases and conditions, which are linked to higher mortality rates [13–15]. It is a serious public health threat that accounts for a significant proportion of the global non-communicable disease burden, including type 2 diabetes, cardiovascular disease, hypertension, and certain cancers [16,17].

Recognizing the responsible factors is pivotal as it enables pre-pregnancy interventions to enhance perinatal outcomes and mitigate associated morbidity and mortality [18]. Certain studies have indicated a notable association between obesity and PAS [19,20], while others have not reported a relationship [21,22], sparking controversy surrounding this matter.

To date, only one meta-analysis has investigated the correlation between obesity and the susceptibility to PAS. The results from this analysis suggest that obesity did not notably elevate the risk of abnormally invasive placenta (OR: 1.37; 95% CI: 1.04, 1.81) [23]. However, its worth noting that this meta-analysis included five studies conducted until February 2017, potentially introducing bias. Hence, we undertook a new meta-analysis to explore the association between obesity and the risk of PAS.

## 2 Materials and methods

Our meta-analysis was conducted following the 2020 Preferred Reporting Items for Systematic Reviews statement.

### 2.1 Eligibility criteria

Inclusion criteria were primary studies evaluating the link between obesity and PAS risk, encompassing various observational study designs such as cohort, case–control, and cross-sectional studies. Obesity was defined as a BMI exceeding 30 kg/m², in accordance with the World Health Organization classification [24]. Exclusion criteria included studies lacking sufficient data for outcome assessment. Additionally, we omitted letters to the editor, case reports, case series, systematic reviews, as well as *in vitro* and animal studies from our analysis.

### 2.2 Information sources and search

A systematic search was conducted on PubMed, Web of Science, Google scholar, and Scopus databases up to March 30, 2024. The search utilized combinations of keywords: (morbidly adherent placenta OR placenta accreta OR placenta increta OR placenta percreta OR abnormally invasive placenta) AND (obesity OR obese) (Table A1). Furthermore, reference lists were scrutinized to identify any additional relevant sources. Gray literature (such as research projects and theses) was examined to find relevant articles.

### 2.3 Study selection

We utilized the population, exposure, comparison, and outcome model to determine eligibility criteria for the studies. The population included pregnant women, with exposure being obesity, compared with a BMI <25 kg/m². The outcome of interest was PAS. Two investigators (E.J. and R.N.) independently screened all titles and abstracts, subsequently reviewing the full texts deemed definitely or possibly eligible. Any discrepancies between the two authors were resolved through discussion.

### 2.4 Data extraction

Information from the included studies was collected using Stata software. This data included details such as the first author, year of article publication, study design, diagnosis criteria, population characteristics, maternal age (in years), and any control measures for confounding variables.

### 2.5 Methodological quality

We utilized the modified Newcastle–Ottawa scale (NOS) to evaluate the quality of observational articles. The NOS comprises three main components: participant selection, comparability of PAS among pregnant women and non-PAS groups, and outcome assessment. Scores on this scale range from 0 to 9, with higher scores indicating better quality. Scores falling between 0 and 6 were classified as low quality, whereas scores ranging from 7 to 9 were categorized as high quality [25].

### 2.6 Heterogeneity and reporting biases

To assess heterogeneity among studies, we employed the chi-square test [26] and the *I* ^2^ statistic [27]. An *I* ^2^ statistic exceeding 50% was interpreted as substantial heterogeneity [27]. Furthermore, regression tests, including Egger’s and Begg’s [28], were utilized to explore publication bias. If *p*-values for Begg’s and Egger’s regression were more than 0.05, there was no evidence of publication bias among the included studies.

### 2.7 Summary measures

The outcomes were categorized as dichotomous variables (PAS compared with non-PAS), and the odds ratio (OR) was presented along with 95% confidence intervals (95% CIs). The analysis was carried out utilizing a random-effects model [29]. If there is heterogeneity in the studies based on these indicators, the random effects model is used. Statistical significance was defined as a *p*-value less than 0.05, and the analysis was performed using Stata software, version 14 (StataCorp, College Station, TX, USA).

## 3 Results

### 3.1 Description of studies

**Figure 1:**
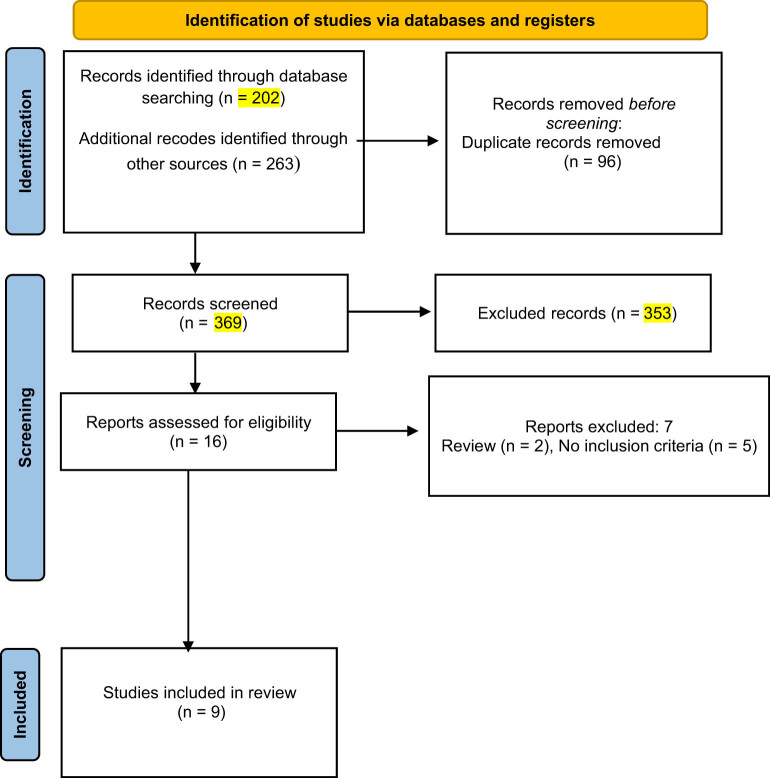
Flowchart of the process selection of the studies.

Initially, we evaluated 465 titles and abstracts, 96 duplicate articles were removed and 369 titles and abstracts were screened, leading to the thorough examination of 16 full papers until April 20, 2024 (Figure 1). Eventually, nine studies met the criteria for inclusion in the current systematic review and meta-analysis. Seven studies were excluded, including two systematic review and five studies that did not meet the inclusion criteria. We identified six studies with a cohort design [10,20–22,30] and three studies with a case–control design [19,31,32]. All studies were published in English. The total number of publication across the included studies was 643,005 (Table 1). The number of patients with PAS was 1,352, and the number of patients with both PAS and obesity was 268.

**Table 1:** Characteristics of the included studies in the present meta-analysis

First author (year)	Design	Sample	Mean of maternal age (year)	Diagnosis criteria	Estimate	Adjustment	Quality
Vieira et al. (2021) [20]	Cohort	386	Case: 34.6 ± 4.7	An expert in a specialist center for PAS according to a strict set of predefined clinical criteria	OR	Crude/adjusted	High
			Control: 34.5 ± 4.7				
Lyell et al. (2015) [19]	Case–control	736	18–34	ICD-9 codes	OR	Crude	High
Ueno et al. (2014) [21]	Cohort	65	Median:35	Pathologically	OR	Crude	High
Eshkoli et al. (2013) [22]	Cohort	34,567	Not reported	Clinical and histopathological reports	OR	Crude	High
Fitzpatrick et al. (2012) [31]	Case–control	390	Not reported	Histologically	RR	Crude/adjusted	High
Thurn et al. (2016) [40]	Cohort	605,567	Not reported	Ultrasound and MRI	OR	Crude	High
Kayem et al. (2024) [10]	Cohort	396	Not reported	Standardized clinical and histological criteria	OR	Crude/adjusted	High
Elbery et al. (2020) [30]	Cohort	33	31.33 ± 5.21	Ultrasonography and histological diagnosis	OR	Crude	Low
Farquhar et al. (2017) [32]	Case–control	865	21–55	Clinical and histological confirmation	OR	Crude	High

### 3.2 Effects of exposure

**Figure 2:**
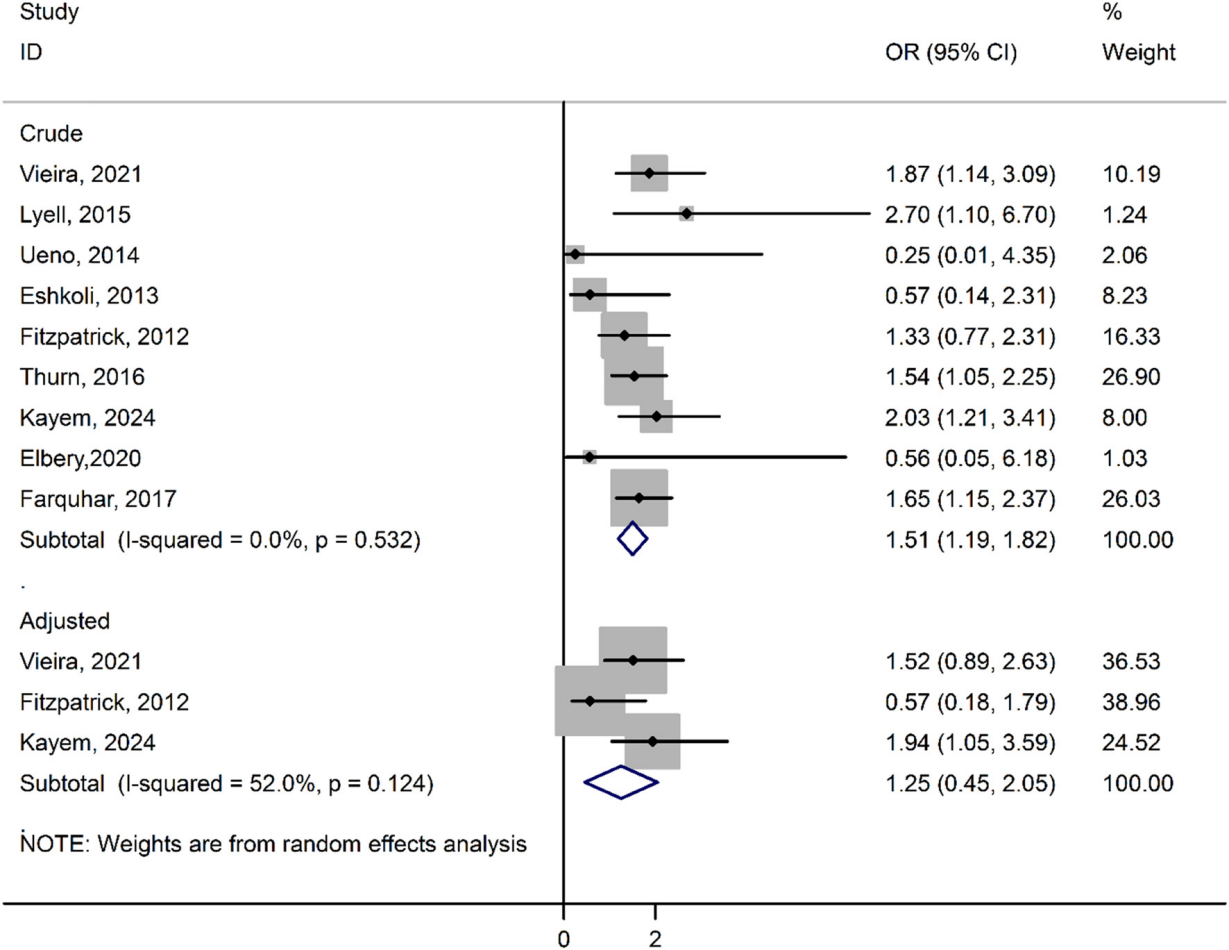
Association between obesity and risk of PAS.

In Figure 2, we investigated the association between obesity and the risk of PAS, considering both crude and adjusted studies. The association between obesity and PAS risk in crude studies showed significance (1.51 [95% CI: 1.19, 1.82; *I* ^2^ = 0.0%]). However, in adjusted studies, the association was not significant (1.25 [95% CI: 0.45, 2.05; *I* ^2^ = 52.0%]). Notably, high heterogeneity was observed among studies based on adjusted analyses, while crude studies demonstrated homogeneity (Figure 2).

### 3.3 Subgroup analysis

Subgroup analysis was performed based on the study design among crude studies. The association between obesity and PAS risk in cohort studies was significant (1.44 [95% CI: 0.96, 1.92; *I* ^2^ = 15.1%]). Conversely, in case–control studies, the association was not significant (1.56 [95% CI: 1.09, 2.03; *I* ^2^ = 0.0%]). Low heterogeneity was observed among cohort studies.

#### 3.3.1 Publication bias

Based on the results of Begg’s and Egger’s tests, we conducted an assessment for publication bias. The *p*-values for Begg’s and Egger’s regression were 0.211 and 0.137, respectively. Hence, there was no evidence of publication bias among the included studies.

#### 3.3.2 Quality of the studies

According to the NOS scale, all studies except for one were rated as high quality (Table 1).

## 4 Discussion

These findings suggest that obesity elevates the risk of PAS in crude studies, whereas in adjusted studies, the association was not significant. High heterogeneity was noted among studies based on adjusted analyses, whereas crude studies exhibited homogeneity.

A sole meta-analysis has been undertaken concerning the association between obesity and the risk of PAS. The results of this analysis reveal a significant increase in the risk of abnormally invasive placenta due to obesity (OR: 1.37; 95% CI: 1.04, 1.81) [23]. Notably, this meta-analysis included five studies conducted until February 2017, with no search conducted in the Web of Science database. Nonetheless, these methodological choices may introduce bias.

Obesity may potentially contribute to abnormal decidualization through various mechanisms, including disruptions in hormonal balance and the impact of endometrial free fatty acid accumulation and lipotoxicity [33]. Another hypothesis posits that the underlying mechanism linking obesity to PAS involves poor wound healing, leading to an increased risk of infection [34]. However, given the non-significant results in the adjusted studies, these hypotheses need to be interpreted with caution.

Women with obesity already face an elevated risk of numerous obstetric complications, including preeclampsia, gestational diabetes, venous thromboembolism, and the necessity for cesarean section delivery, as well as heightened neonatal morbidity and mortality [35]. A recent synthesis of evidence regarding the perception of pregnancy risks among women with obesity revealed that they often felt fearful during consultations due to the perceived stigma associated with obesity, which consequently led to an overestimation of the risks [36]. However, given that obesity is a risk factor for PAS, physicians and healthcare providers are encouraged to advocate for pre-pregnancy counseling to address women’s fitness concerns.

There are hemorrhagic and surgical risks potentially associated with PAS concomitant with obesity. Various treatment methods exist. A multidisciplinary pathway, including a single-surgery protocol with multivessel uterine embolization, is associated with a decrease in blood transfusion requirements and estimated blood loss, without an increase in operative complications. The PAS treatment with intraoperative multivessel embolization protocol provides a definitive surgical method that warrants consideration by other centers specializing in PAS treatment [37]. Also, tranexamic acid has been suggested for preventing or treating primary postpartum hemorrhage, which is the leading cause of maternal morbidity and mortality worldwide [38].

This study’s strengths include the absence of evidence of publication bias among the included studies and the majority of these studies (with the exception of one) being of high quality. Furthermore, the results demonstrated considerable homogeneity among the crude studies.

The implications of these findings for clinical practice include the prevention of obesity in women who want to become pregnant. Therefore, maintaining fitness can reduce the risk of PAS.

This study encountered two limitations. First, there was notable heterogeneity among the adjusted studies, although caution is warranted when interpreting statistical tests for heterogeneity. The *Q*-test has low power when the number of studies in a meta-analysis is low [39], which constituted a limitation of the present meta-analysis. Second, only three studies were adjusted for confounders, potentially resulting in information bias. Nonetheless, despite these limitations, this meta-analysis, which encompassed 643,005 subjects, suggests that previous abortion heightens the risk of PAS.

## 5 Conclusion

These findings indicate that obesity has been proposed as being related with a higher danger of PAS among crude studies. However, it is necessary to conduct prospective cohort studies with a large sample size and control of confounding variables.

## Appendix

**Table A1:** Strategy search

**PubMed**
("obesity"[MeSH Major Topic] OR "obesity"[MeSH Major Topic] OR "obesity"[MeSH Major Topic]) OR " obese*"[MeSH Major Topic]) AND ("morbidly adherent placenta"[Title/Abstract] OR "placenta accreta"[Title/Abstract] OR "placenta increta"[Title/Abstract] OR "placenta percreta"[Title/Abstract] OR "abnormally invasive placenta"[Title/Abstract] OR "placenta accreta"[MeSH Major Topic])
**Scopus**
((TITLE-ABS-KEY (obese)) OR (TITLE-ABS-KEY (obesity))) AND ((TITLE-ABS-KEY (morbidly AND adherent AND placenta)) OR (TITLE-ABS-KEY (placenta AND accreta)) OR (TITLE-ABS-KEY (placenta AND increta)) OR (TITLE-ABS-KEY (placenta AND percreta)) OR (TITLE-ABS-KEY (abnormally AND invasive AND placenta)))
**Web of Science**
(TS=(obesity)) OR TS=(obese) AND ((((TS=(morbidly adherent placenta)) OR TS=(placenta accreta )) OR TS=(placenta increta )) OR TS=(placenta percreta )) OR TS=(abnormally invasive placenta))
